# The Intersection of Neuroscience and Criminology: Role of Brain Mapping in Addressing Criminal Behavior by Nepal Police

**DOI:** 10.31729/jnma.8786

**Published:** 2024-10-31

**Authors:** Bibek Rajbhandari, Sushant Regmi, Kanchan Dahal, Sandesh Shrestha

**Affiliations:** 1 Department of General Practice and Emergency Medicine, Nepal Police Hospital, Maharajgunj, Kathmandu, Nepal; 2 Department of General Practice and Emergency Medicine, Patan Academy of Health Sciences, Lagankhel, Lalitpur, Nepal; 3 Central Jail Hospital, Sundhara, Kathmandu, Nepal; 4 District Government Attorney Office, Kathmandu, Nepal

**Keywords:** *brain mapping*, *criminology*, *Nepal*, *police*

## Abstract

Neuroscience is being integrated into criminal investigations, offering new opportunities for understanding, predicting, and potentially preventing criminal behavior. In Nepal, brain-mapping techniques like P300 wave tests and Brain Electrical Oscillation Signature profiling were used in a murder investigation in 2024. However, ethical concerns include privacy concerns, false positives, and the potential for false positives. Consent regarding neurobiological evidence in criminal cases is crucial, as intrusive procedures may require individuals to be informed. The legal implications of using neurobiological evidence could be severe if it serves to wrongfully accuse or convict an individual. Therefore, the use of neuroscience in criminology must be carefully balanced to ensure ethical considerations. This viewpoint interprets the potential of neuroscience in criminal investigations, addressing ethical, privacy, consent, and legal issues within the Nepal Police and criminal justice system.

## INTRODUCTION

Criminal and antisocial behaviors pose considerable social and security risks, which certainly raise the demand for new approaches to mitigate the problem.^1^ This fact makes it imperative for society to find a solution by using an innovative approach to counter criminal and antisocial behaviors. Over the last two decades, studies related to neuroscience have considerably enlightened us about the brain processes involved in criminal behavior.^2^ These developments made it possible to use neuroscience-based strategies in the legal and judicial sciences. In fact, the identification of biological markers in the neurobiology that correlates with antisocial and violent acts has become a promising development.^3^ It is these developments within neurocriminology that are giving police and researchers new tools and methods to study, forecast, and perhaps prevent such behavior.

**Biological and Neurological Factors in Criminology**

The biological theory in the field of criminology looks into how various physiological and neurological factors culminate into criminal behavior.^4-6^ This theory is based on the "born criminal" theory of Cesare Lombroso, who believed that certain physical features are indicative of criminal tendencies.^7^ This has been shrouded by much criticism, but it formed a basis for understanding how the workings of the brain constitute behavior. Brain mapping has identified how areas of the brain, including the prefrontal cortex and the amygdala, influence behavior.^8^ Tumors, lesions, and trauma to such sites have been associated with impulsive behavior, violence, and antisocial conduct.^9,10^ Neurotransmitters, such as serotonin and dopamine, and hormones like cortisol and testosterone also interact in the control of mood and aggression.^11^ Further intertwining the biological and environmental aspects is the so-called "warrior gene"-MAOA-which may offer some potential keys to prevention and intervention in the study of criminology.

**Applications of Brain-Mapping Technologies in Criminal Investigations**

Brain mapping is one of the promising technologies in this area and involves the testing for a P300 wave. It is used to record the brain activities emitted in response to certain images or words to decide on any person's knowledge about a case.^12,13^ Another kind of technique of brain fingerprinting is "Brain Electrical Oscillation Signature (BEOS) profiling can also distinguish between witnesses and perpetrators" it has been used in more than 1000 forensic cases.^14^ This technique is done to find what information a suspect has about a crime in the case of some memories of experiences. The suspect is exposed to three categories of words: one which does not relate directly to the case, probe words in the second category, which are directly related to the case, and the target which is formulated on the basis of the confidential findings.^15^ The result of using BEOS profiling was revolutionary and gave the solid evidence to hook up the criminal behavior to his brain activities. This new methodology replaced the traditional way of doing investigation and the finding of a suspect became more accurate and time-effective. Now the use of brain mapping on criminal activities is extremely appreciated and adopted by almost all countries' police departments, especially in India, where the judgment on the decision to use BEOS as in the case of Maharashtra and the Chembur case.^16^

**The First Instance of Brain Mapping Using BEOS Profiling in a Landmark Murder Investigation**

In early 2024, Nepal witnessed a groundbreaking development in applying brain mapping to criminal investigations. BEOS profiling was first used on a murder investigation; it indicated that the suspect had knowledge of the crime. Such technology in legal processes has sparked controversy over the scientific validity and the ethical concerns. BEOS test sensitivity and specificity during the study conducted by Technology Information Forecasting and Assessment Council-TIFAC, India, New Delhi, and Directorate of Forensic Science, Gandhinagar had achieved 95% and 94%, respectively. At a cutoff set at 3 standard deviations from the scores of the control group, specificity increased to 100%.^15^ That is, it did not mislabel any innocent people as involved. Critics say that the technology violates privacy and will catch too many false positives, while supporters claim it has its value in evidence that might be impossible for traditional techniques to obtain. As BEOS profiling continues to be employed in criminal investigations, the debate over its reliability and ethical implications will likely persist.

**The Intersection of Science and Law**

This branch of science is developing, and with every step in its development, it finds its application in the legal system one way or another. Though the legal system of Nepal depends on conventional methods of investigation, brain mapping has found its way globally and is gaining momentum with every step forward; therefore, this might affect criminal justice in the near future. The use of brain imaging evidence in criminal cases, particularly in capital trials in countries like the United States, has been very guarded.^17^ This cautious reception is based on various ethical issues and questions over reliability. European courts have been even more conservative, while countries like India and China have invested heavily in neuroscience despite ongoing debates about the admissibility and ethics of such evidence.^18,19^

**Limitations of Brain Mapping Technique**

However, the technique of brain mapping is not devoid of certain drawbacks. The most significant one is that it is totally dependent on the subject for the familiarity of information pertaining to the crime on account of external factors such as media exposure or incidental information. To put it differently, the identification of a P300 wave or its analog in brain activity may not per se be identified with guilt or involvement in a crime. It is also followed by ethical problems with the aspect of misuse, intrusion of privacy, and some give false positives, which may lead to convictions of innocents.

## WAY FORWARD

The integration of neuroscience into criminal justice demands a multidimensional approach. This involves creating accuracy and dependability of brain mapping techniques such as BEOS profiling and the P300 wave test in its development to establish standardized protocols that generate consistent results, most importantly, with high specificity of the tests if not 100% to ensure no innocent person is labeled guilty. This will, in addition, help to manage the privacy concerns and regulate neuroscience evidence in court through the drawing up of stringent ethical guidelines and legal yardsticks. Increased collaboration by neuroscientists, legal experts, and ethicists, along with more comprehensive training and education, will foster responsible use and interpretation of these technologies. Furthermore, studies on the brain in relation to crime have to be pursued to find their implications for society and the public's discursive activity. In this way, scientific progress and ethics would be balanced, so that brain mapping developments enhance justice without compromising individual rights.
